# Supplementary material for: Integration of time-series meta-omics data reveals how microbial ecosystems respond to disturbance
Source: Nat Commun. 2020 Oct 19;11:5281. doi: 10.1038/s41467-020-19006-2 (PMC7572474; doi:10.1038/s41467-020-19006-2)
Supplement: Supplementary file 1 — Supplementary Information [file 41467_2020_19006_MOESM1_ESM.pdf]

Supplementary Information

Integration of time-series meta-omics data reveals how microbial ecosystems respond to disturbance

Herold et al.

Table of Contents

Supplementary Figures 2

Supplementary Note 1: Expression of key functions involved in lipid metabolism over time *18*

Supplementary References *20*

Supplementary Figures

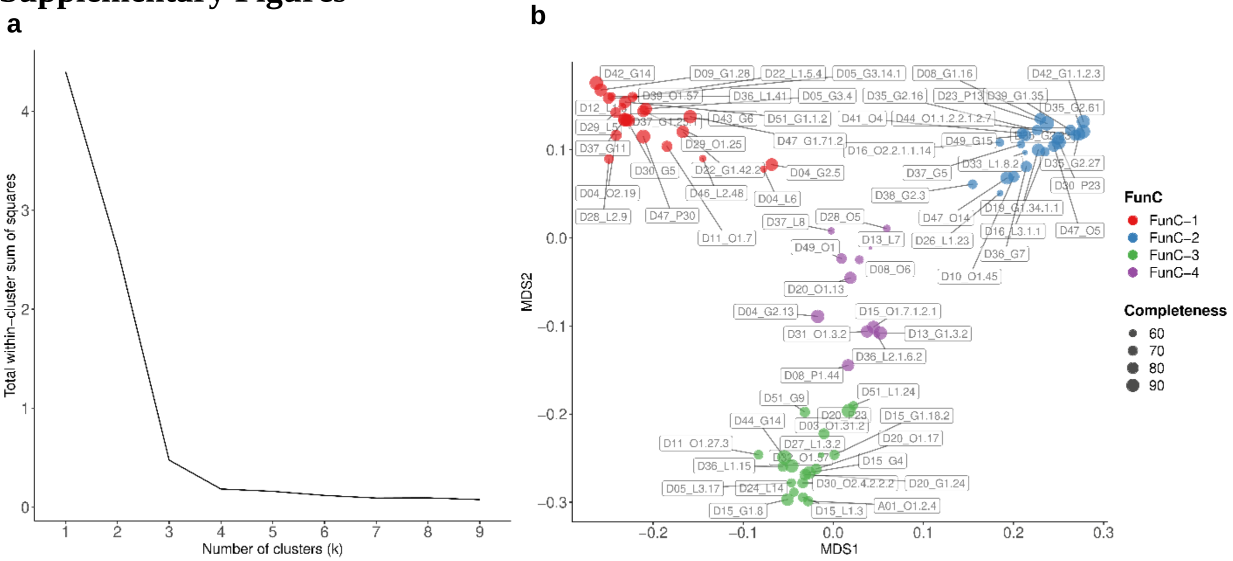

Supplementary Figure 1: Clustering of functional potential. (**a**) Total within-cluster sum of squares shown for different values of k in the k-means clustering of KEGG ortholog presence/absence profiles of the rMAGs. (**b**) Assignment of clusters according to k-means clustering with k=4 with colour indicating the assigned cluster. Labels indicate rMAG-identifier and point size reflects MAG completeness as estimated with CheckM.

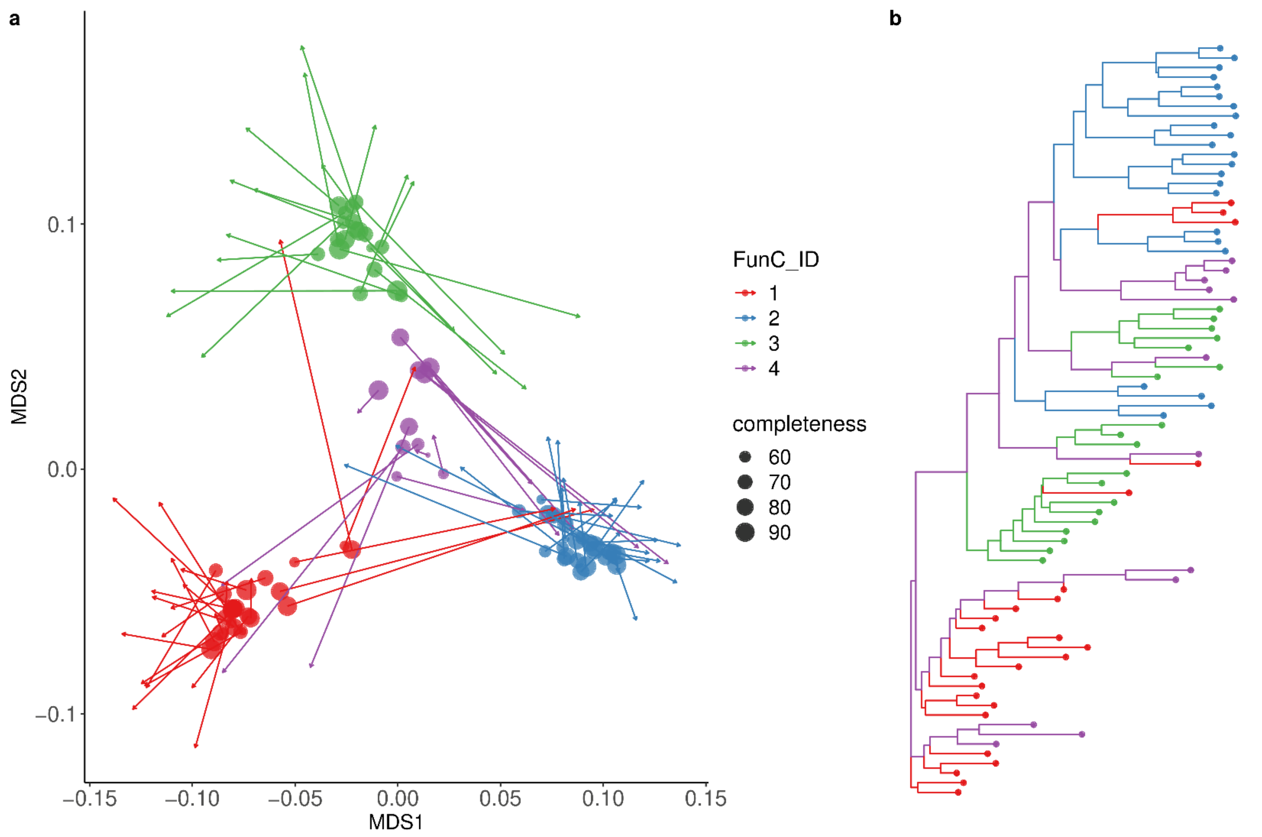

Supplementary Figure 2: Procrustes analysis comparing an embedding of rMAG distances of their KO profiles (see Fig 2a, Supplementary Fig 1b) to an embedding based on genomic distances computed using mash (**a**). Point size indicates rMAG completeness and colour the FunC assignment of an rMAG. Arrows indicate the position of the embedded points in the multi-dimension scaling of genomic distances after rotation and scaling. (**b**) Genomic distances were inferred with mash and are visualised as a neighbour joining based tree.

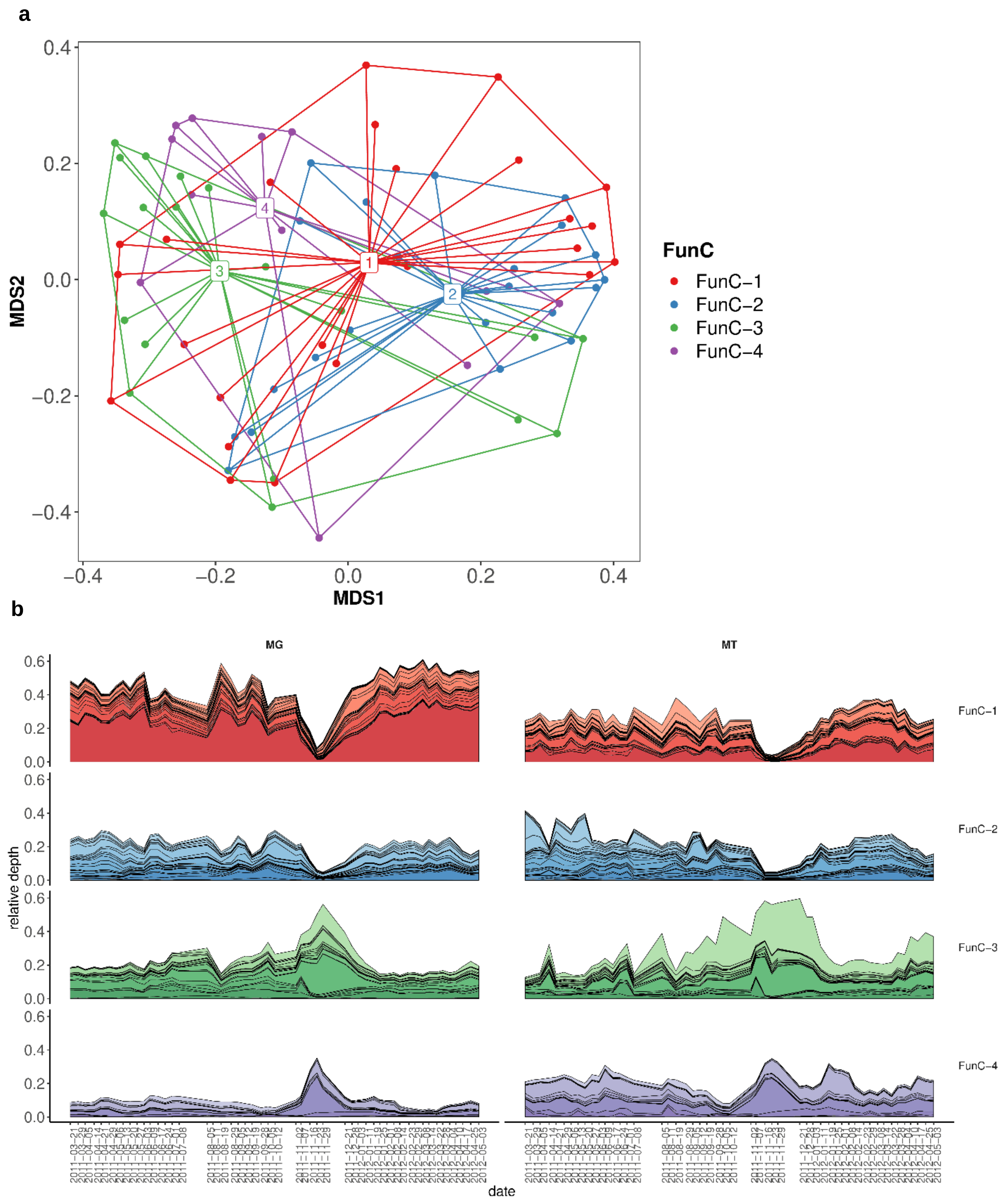

Supplementary Figure 3: FunC dynamics. (**a**) Spider-plot of ordination of distances based on intra-rMAG abundance correlations. Individual rMAGs marked by FunC assignment (color). FunC-centroids are shown and lines connect the rMAGs to their respective FunC-centroid. (**b**) Relative abundance (MG depth) and activity (MT depth) of rMAGs over-time. Selected rMAGs are shown (n=78; see also main text). Color-palettes and separated panels indicate FunC assignment, red: FunC1, blue: FunC2, green: FunC3, purple: FunC-4

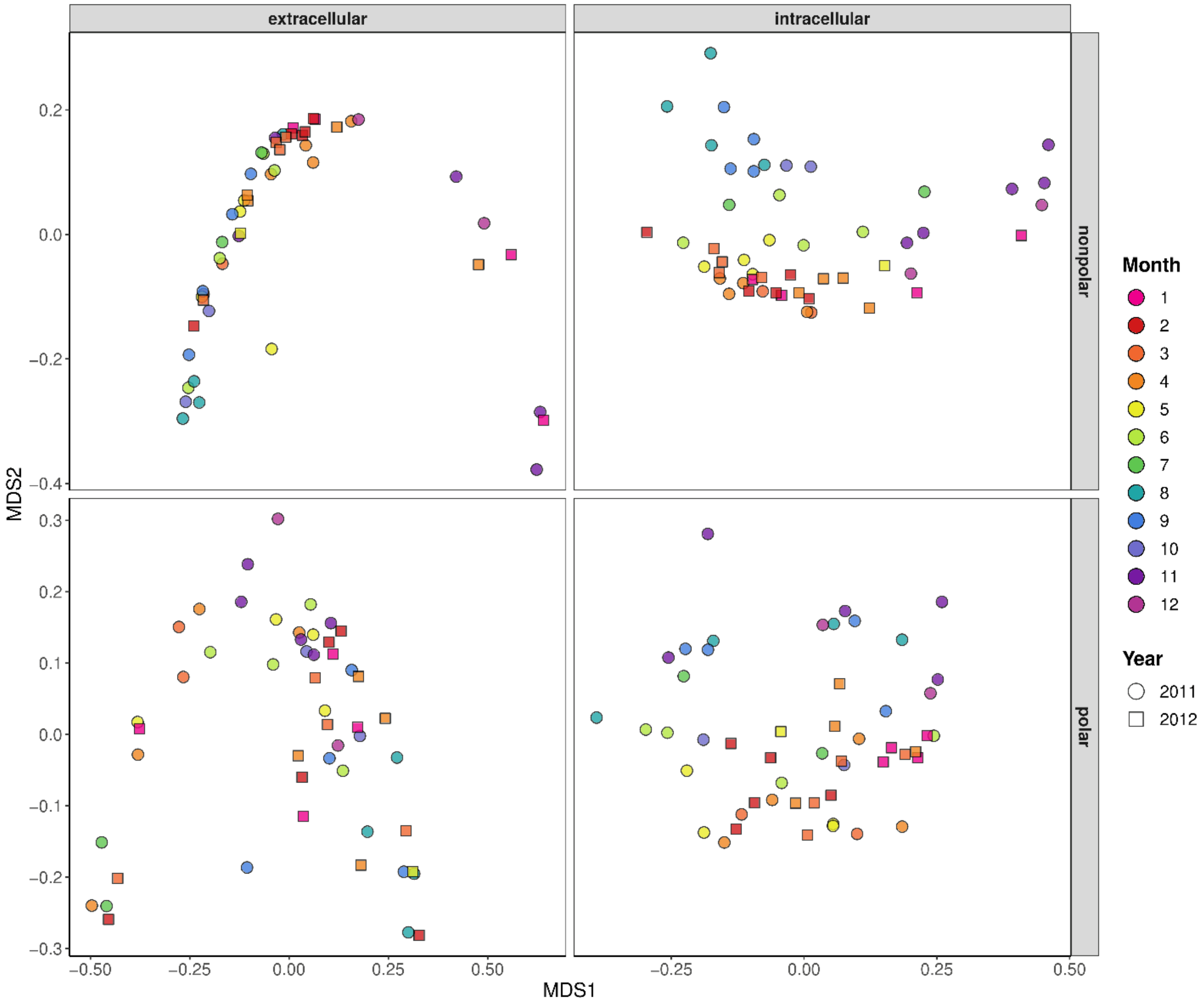

Supplementary Figure 4: Ordination of Bray-Curtis dissimilarity of metabolite intensities. Ordinations for four distinct measurements are shown: nonpolar extracellular (upper left), nonpolar intracellular (upper right), polar extracellular (lower left), polar intracellular (lower right). Colors indicate the month of the sampling date and point-shapes reflect the year. n=51 time-points.

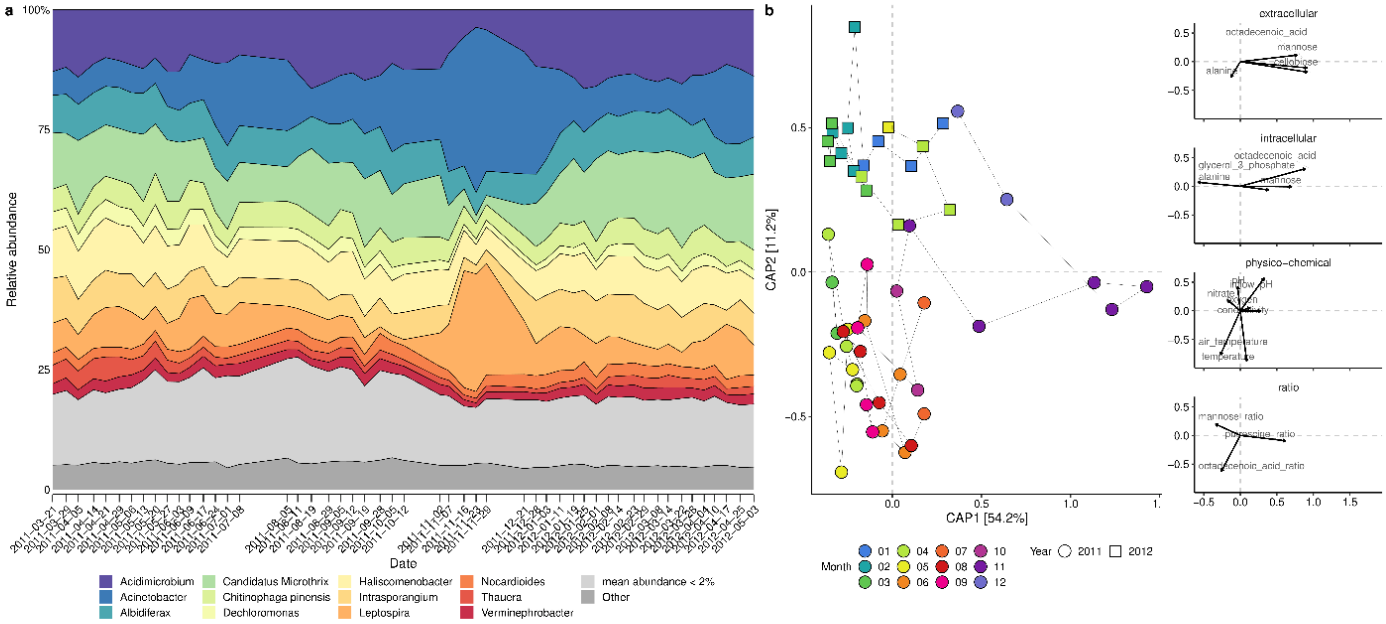

Supplementary Figure 5: (**a)** Relative levels of MP spectral counts for recovered populations (rMAGs). The relative counts of individual rMAGs are grouped based on genus-level taxonomic assignment with rMAGs of unresolved taxonomy grouped in “Other”. Recoverd genera with mean relative counts below 2% are summarized as a single group (light grey). n=78 rMAGs. (**b)** Ordination of Bray-Curtis dissimilarity of the relative counts of individual rMAGs constrained by selected abiotic factors (levels of metabolites, metabolite-ratios, and physico-chemical parameters fit as vectors; black arrows and labelled arrowheads; with arrow length indicating environmental scores as predictors for each factor). Points are coloured by month of sampling and point-shape reflects the year of sampling. Thin black lines visualize the time course of sampling. n=51 time-points.

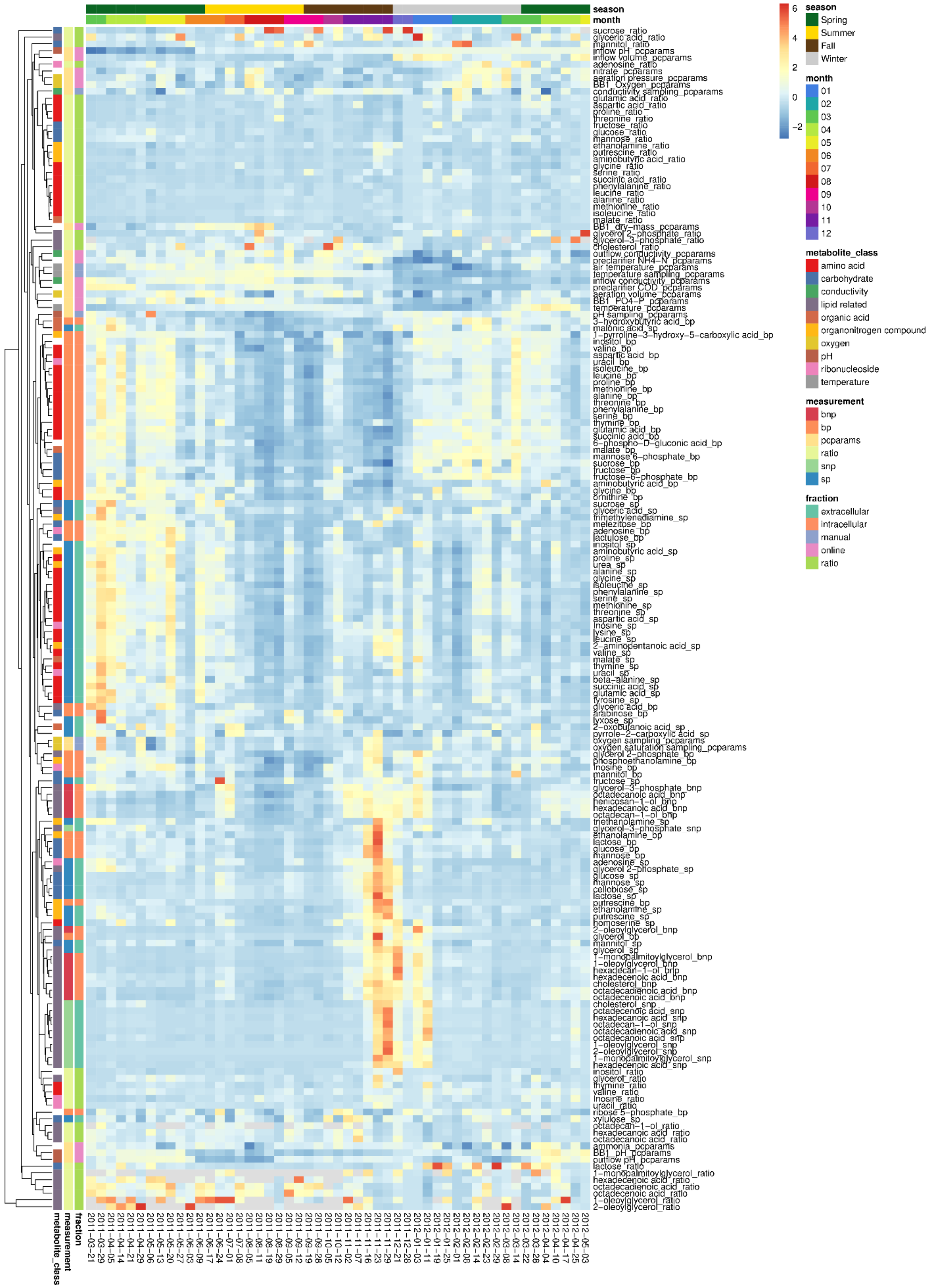
season
month
metabolite_class
measurement
fraction
sucrose_ratio
glyceric acid_ratio
mannitol_ratio
inflow pH_pcparams
inflow volume_pcparams
adenosine_ratio
nitrate_pcparams
aeration pressure_pcparams
BB1_Oxygen_pcparams
conductivity sampling_pcparams
glutamic acid_ratio
aspartic acid_ratio
proline_ratio
threonine_ratio
fructose_ratio
glucose_ratio
mannose_ratio
ethanolamine_ratio
putrescine_ratio
aminobutyric acid_ratio
glycine_ratio
serine_ratio
succinic acid_ratio
phenylalanine_ratio
leucine_ratio
alanine_ratio
methionine_ratio
isoleucine_ratio
malate_ratio
BB1_dry−mass_pcparams
glycerol 2−phosphate_ratio
glycerol−3−phosphate_ratio
cholesterol_ratio
outflow conductivity_pcparams
preclarifier NH4−N_pcparams
air temperature_pcparams
temperature sampling_pcparams
inflow conductivity_pcparams
preclarifier COD_pcparams
aeration volume_pcparams
BB1_PO4−P_pcparams
temperature_pcparams
pH sampling_pcparams
3−hydroxybutyric acid_bp
malonic acid_sp
1−pyrroline−3−hydroxy−5−carboxylic acid_bp
inositol_bp
valine_bp
aspartic acid_bp
uracil_bp
isoleucine_bp
leucine_bp
proline_bp
methionine_bp
alanine_bp
threonine_bp
phenylalanine_bp
serine_bp
thymine_bp
glutamic acid_bp
succinic acid_bp
6−phospho−D−gluconic acid_bp
malate_bp
mannose 6−phosphate_bp
sucrose_bp
fructose_bp
fructose−6−phosphate_bp
aminobutyric acid_bp
glycine_bp
ornithine_bp
sucrose_sp
glyceric acid_sp
trimethylenediamine_sp
melezitose_bp
adenosine_bp
lactulose_bp
inositol_sp
aminobutyric acid_sp
proline_sp
urea_sp
alanine_sp
glycine_sp
isoleucine_sp
phenylalanine_sp
serine_sp
methionine_sp
threonine_sp
aspartic acid_sp
inosine_sp
lysine_sp
leucine_sp
2−aminopentanoic acid_sp
valine_sp
malate_sp
thymine_sp
uracil_sp
beta−alanine_sp
succinic acid_sp
glutamic acid_sp
tyrosine_sp
glyceric acid_bp
arabinose_bp
lyxose_sp
2−oxobutanoic acid_sp
pyrrole−2−carboxylic acid_sp
oxygen sampling_pcparams
oxygen saturation sampling_pcparams
glycerol 2−phosphate_bp
phosphoethanolamine_bp
inosine_bp
mannitol_bp
fructose_sp
glycerol−3−phosphate_bnp
octadecanoic acid_bnp
henicosan−1−ol_bnp
hexadecanoic acid_bnp
octadecan−1−ol_bnp
triethanolamine_sp
glycerol−3−phosphate_snp
ethanolamine_bp
lactose_bp
glucose_bp
mannose_bp
adenosine_sp
glycerol 2−phosphate_sp
glucose_sp
mannose_sp
cellobiose_sp
lactose_sp
putrescine_bp
ethanolamine_sp
putrescine_sp
homoserine_sp
2−oleoylglycerol_bnp
glycerol_bp
mannitol_sp
glycerol_sp
1−monopalmitoylglycerol_bnp
1−oleoylglycerol_bnp
hexadecan−1−ol_bnp
hexadecenoic acid_bnp
cholesterol_bnp
octadecadienoic acid_bnp
octadecenoic acid_bnp
cholesterol_snp
octadecenoic acid_snp
hexadecanoic acid_snp
octadecan−1−ol_snp
octadecadienoic acid_snp
octadecanoic acid_snp
1−oleoylglycerol_snp
2−oleoylglycerol_snp
1−monopalmitoylglycerol_snp
hexadecenoic acid_snp
inositol_ratio
glycerol_ratio
thymine_ratio
valine_ratio
inosine_ratio
uracil_ratio
ribose 5−phosphate_bp
xylulose_sp
octadecan−1−ol_ratio
hexadecanoic acid_ratio
octadecanoic acid_ratio
ammonia_pcparams
BB1_pH_pcparams
outflow pH_pcparams
lactose_ratio
1−monopalmitoylglycerol_ratio
hexadecenoic acid_ratio
octadecadienoic acid_ratio
octadecenoic acid_ratio
1−oleoylglycerol_ratio
2−oleoylglycerol_ratio
2011−03−21
2011−03−29
2011−04−05
2011−04−14
2011−04−21
2011−04−29
2011−05−06
2011−05−13
2011−05−20
2011−05−27
2011−06−03
2011−06−09
2011−06−17
2011−06−24
2011−07−01
2011−07−08
2011−08−05
2011−08−11
2011−08−19
2011−08−29
2011−09−05
2011−09−12
2011−09−19
2011−09−28
2011−10−05
2011−10−12
2011−11−02
2011−11−07
2011−11−16
2011−11−23
2011−11−29
2011−12−21
2011−12−28
2012−01−03
2012−01−11
2012−01−19
2012−01−25
2012−02−01
2012−02−08
2012−02−14
2012−02−23
2012−02−29
2012−03−08
2012−03−14
2012−03−22
2012−03−28
2012−04−04
2012−04−10
2012−04−17
2012−04−25
2012−05−03
6
4
2
0
−2
season
Spring
Summer
Fall
Winter
month
01
02
03
04
05
06
07
08
09
10
11
12
metabolite_class
amino acid
carbohydrate
conductivity
lipid related
organic acid
organonitrogen compound
oxygen
pH
ribonucleoside
temperature
measurement
bnp
bp
pcparams
ratio
snp
sp
fraction
extracellular
intracellular
manual
online
ratio

Supplementary Figure 6: Heatmap of z-score transformed intensities of metabolites, metabolite-ratios (intra-*vs.*-extracellular) and measurements of physico-chemical parameters. Row annotations show the broad type of the metabolite or measurement (metabolite_class), while “measurement” and “fraction” distinguish the individual measurements. Column annotations show the season and month of the sampling dates (n=51 time-points).

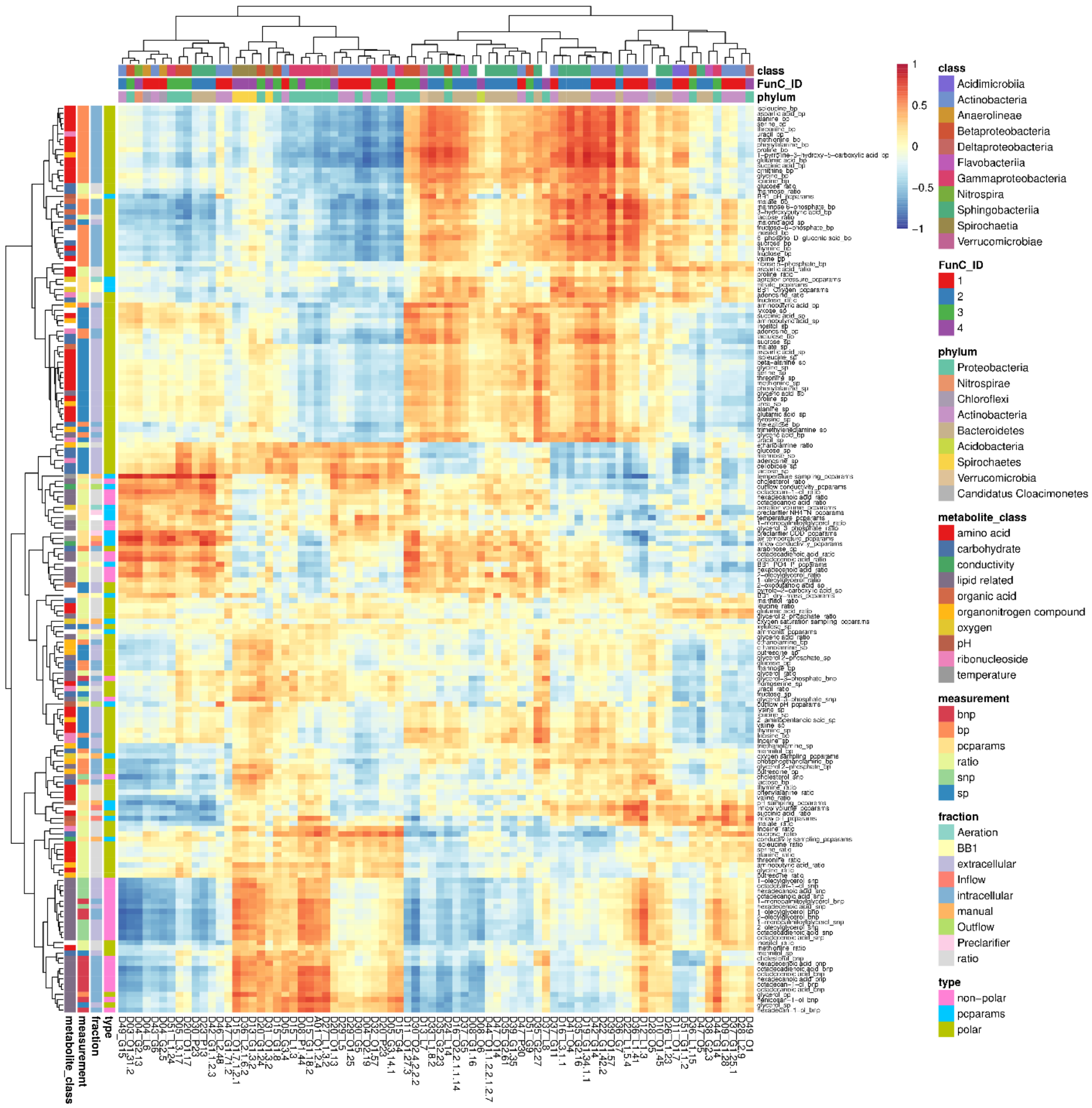

Supplementary Figure 7: Heatmap of pairwise Spearman rank correlations of metabolite or physico-chemical parameter measurements to rMAG relative abundances (n=78 rMAGs). Metabolite values are z-score normalised. Row annotations show information on metabolite measurements while column annotations show rMAG taxonomy or FunC assignment.

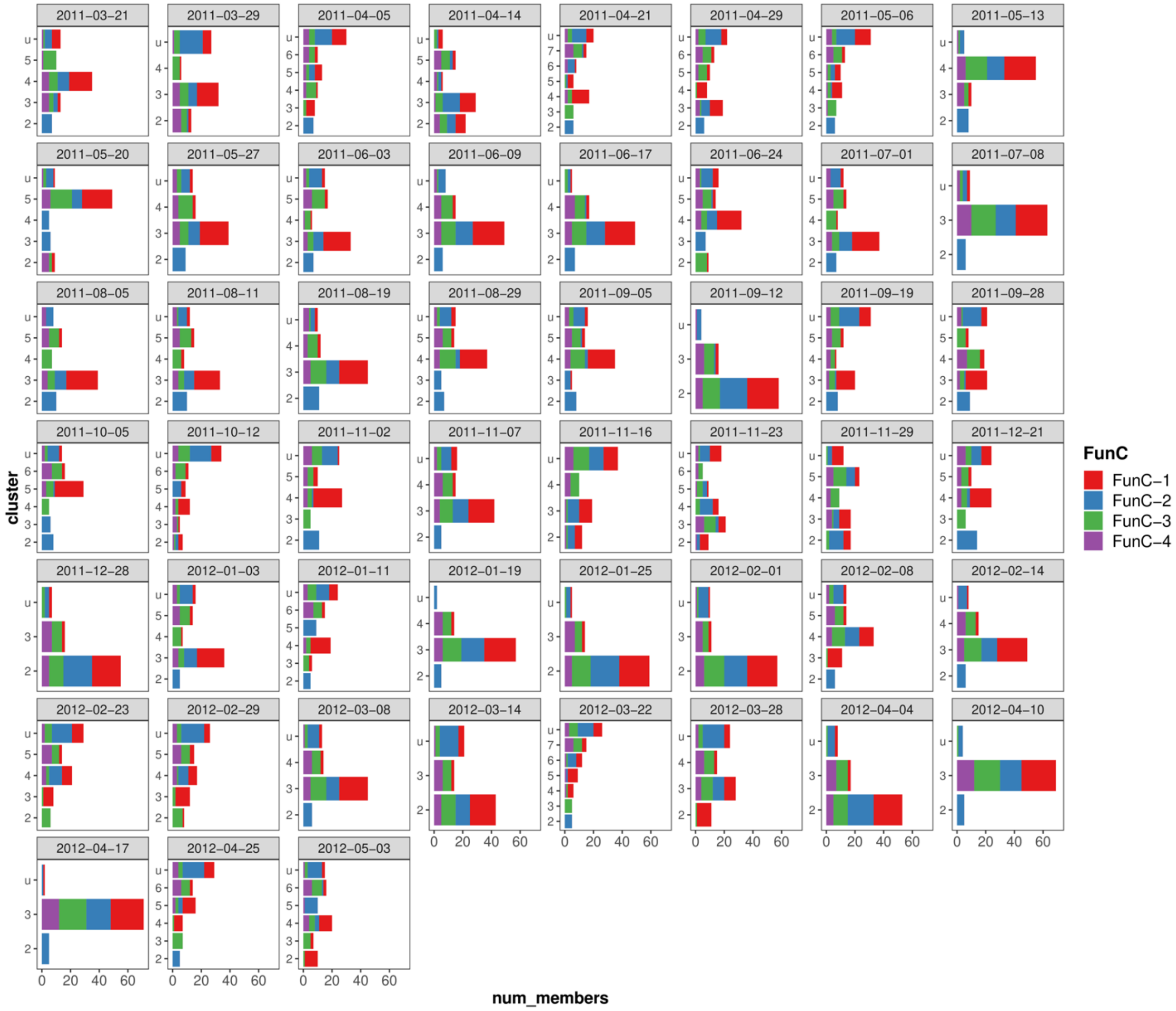

Supplementary Figure 8:

Number of rMAGs (of n=78 rMAGs) assigned to clusters according to expression profiles for every *in situ* time-point. The identifiers of clusters are shown (y-axis) with "u" marking the group of unassigned rMAGs. Colored bars indicate the numbers of rMAGs according to their FunC assignment.

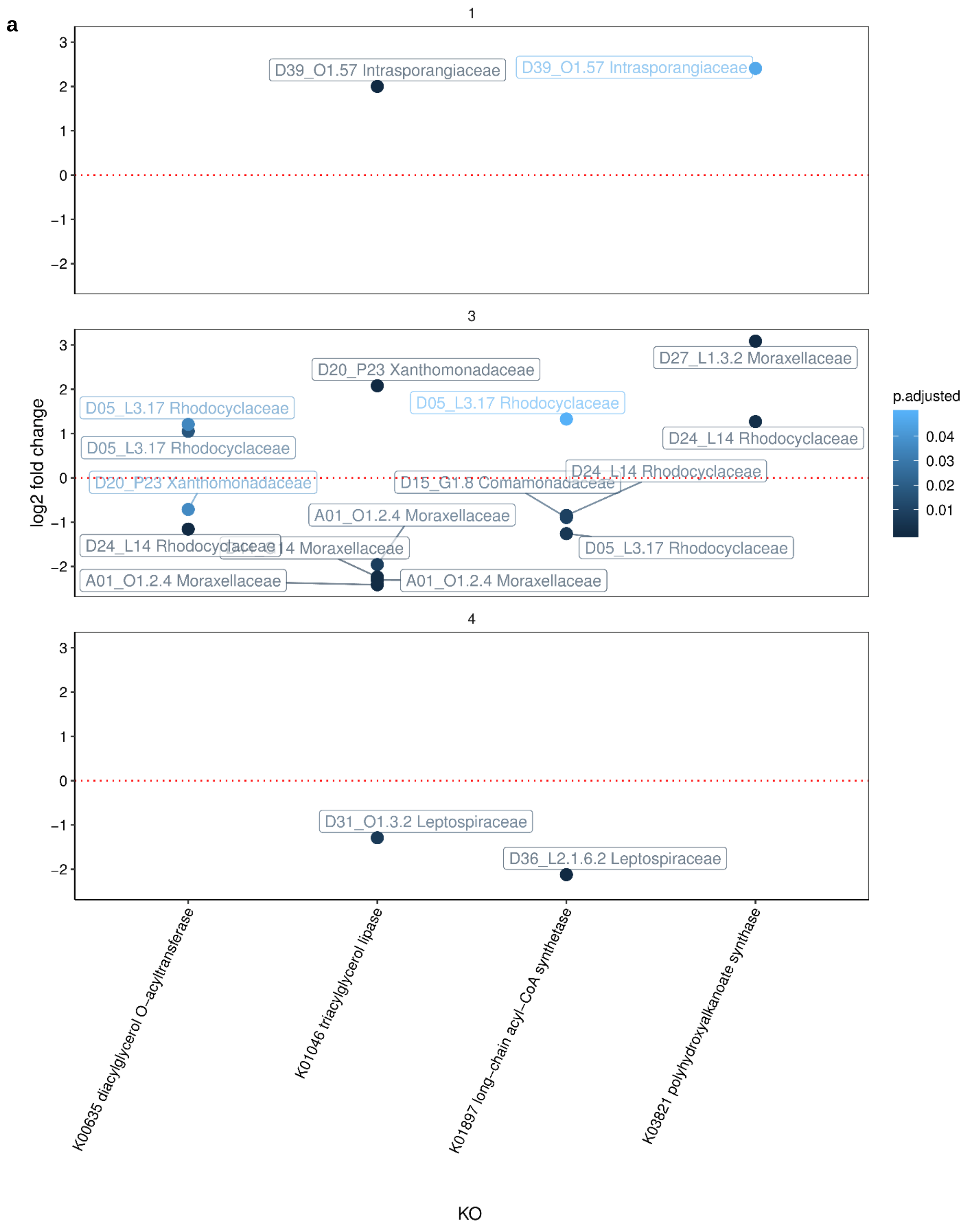
a
1
D39_O1.57 Intrasporangiaceae
D39_O1.57 Intrasporangiaceae
3
D27_L1.3.2 Moraxellaceae
D20_P23 Xanthomonadaceae
D05_L3.17 Rhodocyclaceae
D05_L3.17 Rhodocyclaceae
D24_L14 Rhodocyclaceae
D05_L3.17 Rhodocyclaceae
D24_L14 Rhodocyclaceae
D20_P23 Xanthomonadaceae
D15_G1.8 Comamonadaceae
A01_O1.2.4 Moraxellaceae
D24_L14 Rhodocyclaceae
D05_L3.17 Rhodocyclaceae
A01_O1.2.4 Moraxellaceae
A01_O1.2.4 Moraxellaceae
4
D31_O1.3.2 Leptospiraceae
D36_L2.1.6.2 Leptospiraceae
log2 fold change
p.adjusted
0.04
0.03
0.02
0.01
K00635 diacylglycerol O-acyltransferase
K01046 triacylglycerol lipase
K01897 long-chain acyl-CoA synthetase
K03821 polyhydroxyalkanoate synthase
KO

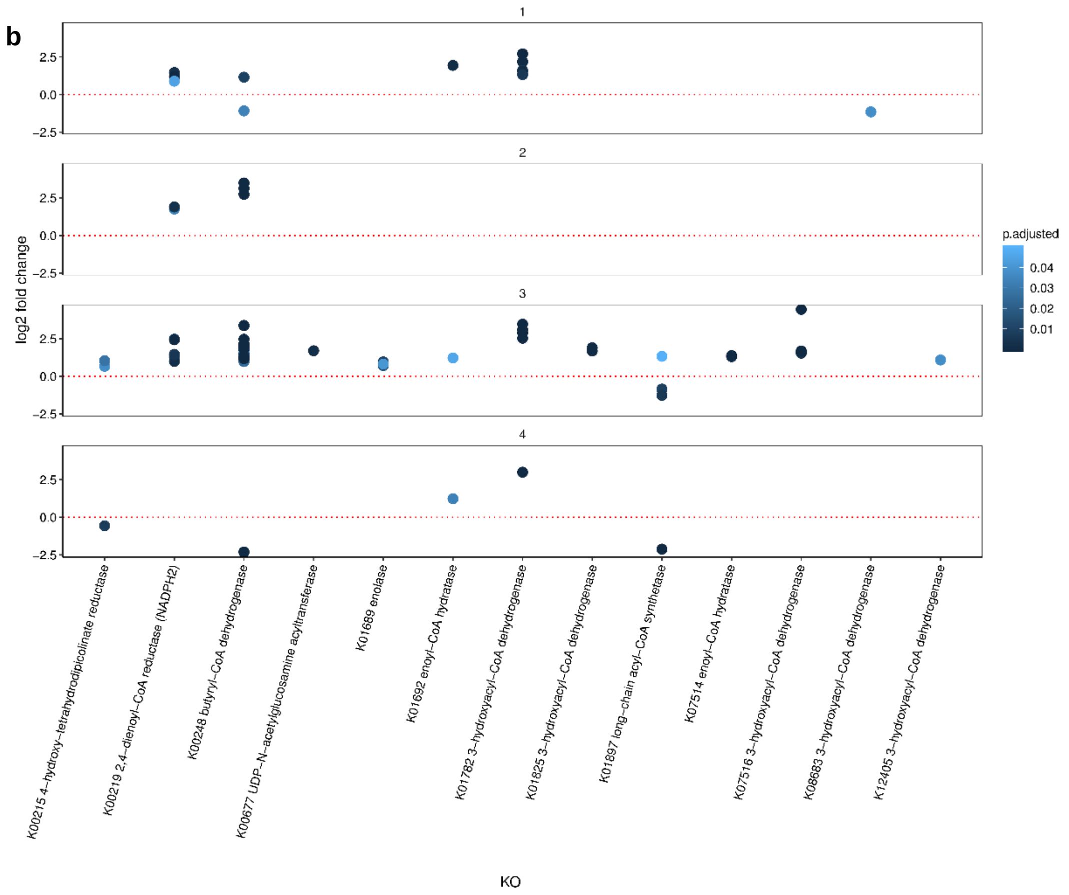

Supplementary Figure 9: Differentially expressed genes (DEGs) in the ex situ experiments comparing 0 h and 5 h time-points. Labels indicate rMAG-identifier and family assignment. The color gradient indicates the adjusted p-value (two-sided Wald test, multiple testing correction with Benjamini & Hochberg method in DESeq2). Individual panels show individual FunCs (1 to 4). Highlighted DEGs were selected for KOs with functions involved in lipid accumulation (**a**) and functions involved in beta-oxidation (**b**).

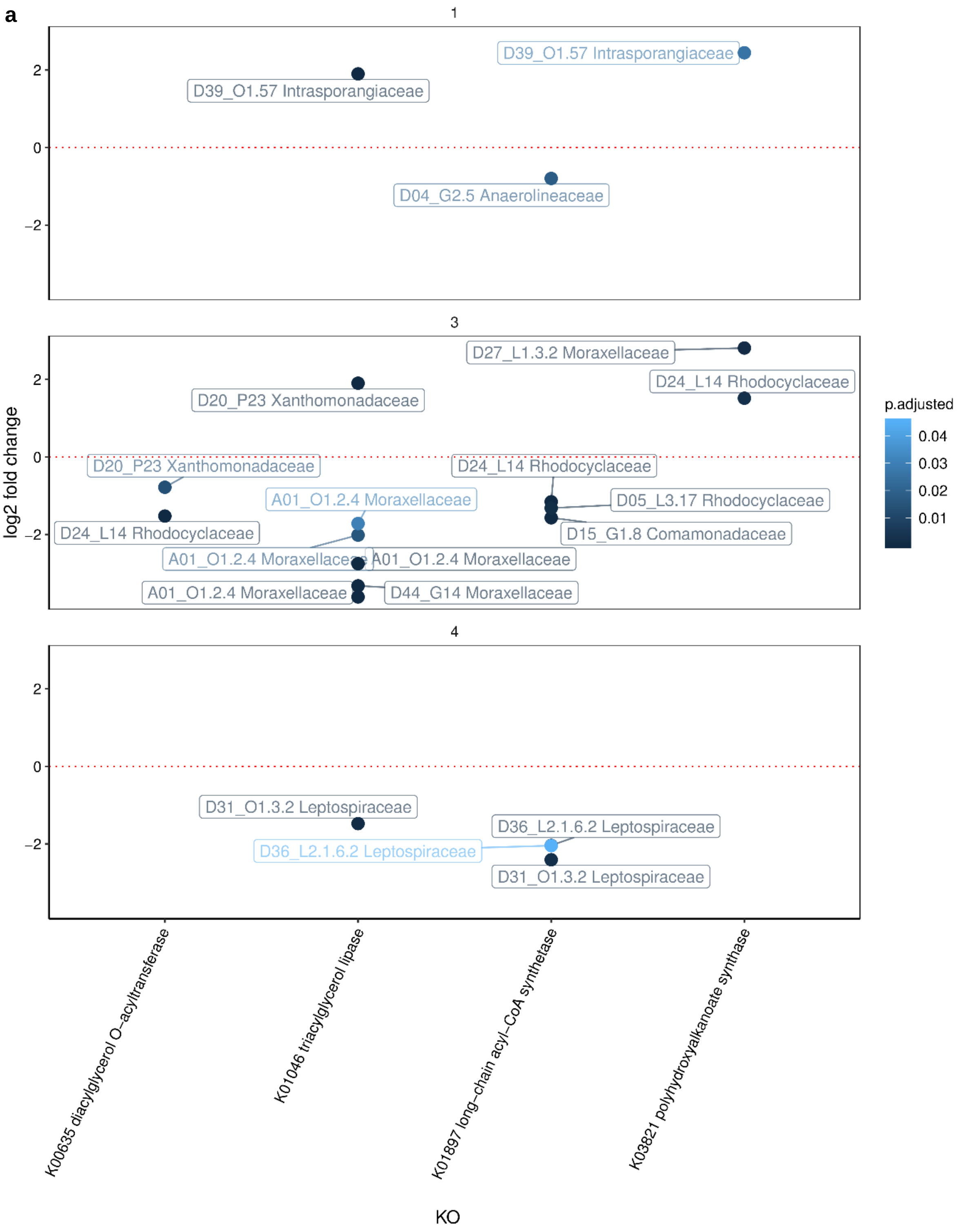

a
1
D39_O1.57 Intrasporangiaceae
D39_O1.57 Intrasporangiaceae
D04_G2.5 Anaerolineaceae
3
D27_L1.3.2 Moraxellaceae
D24_L14 Rhodocyclaceae
D20_P23 Xanthomonadaceae
D20_P23 Xanthomonadaceae
D24_L14 Rhodocyclaceae
A01_O1.2.4 Moraxellaceae
D05_L3.17 Rhodocyclaceae
D24_L14 Rhodocyclaceae
D15_G1.8 Comamonadaceae
A01_O1.2.4 Moraxellaceae
A01_O1.2.4 Moraxellaceae
A01_O1.2.4 Moraxellaceae
D44_G14 Moraxellaceae
4
D31_O1.3.2 Leptospiraceae
D36_L2.1.6.2 Leptospiraceae
D36_L2.1.6.2 Leptospiraceae
D31_O1.3.2 Leptospiraceae
log2 fold change
p.adjusted
0.04
0.03
0.02
0.01
K00635 diacylglycerol O–acyltransferase
K01046 triacylglycerol lipase
K01897 long–chain acyl–CoA synthetase
K03821 polyhydroxyalkanoate synthase
KO

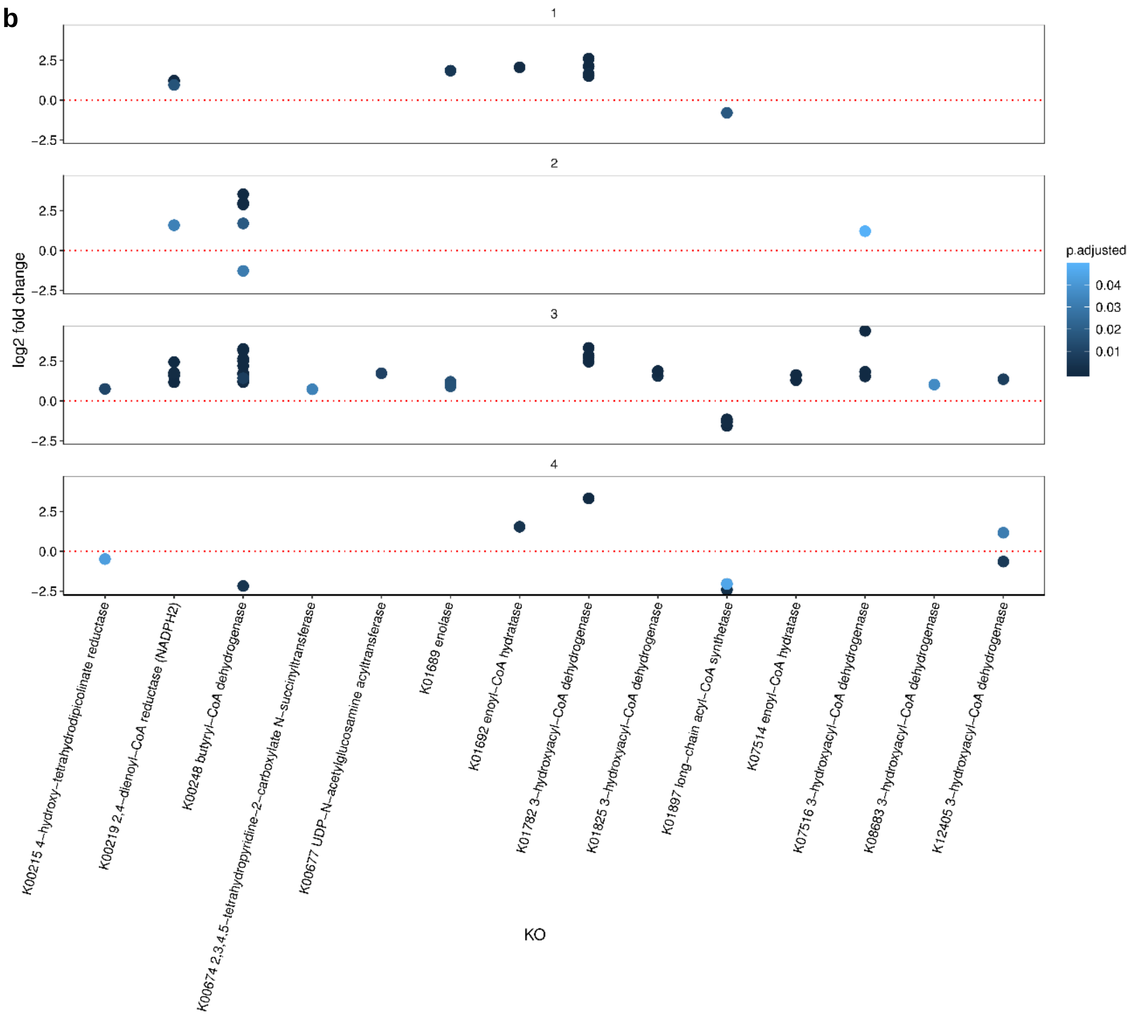

Supplementary Figure 10: Differentially expressed genes (DEG) in the ex-situ experiments 0 h and 8 h time-points. Labels indicate rMAG-identifier and family assignment. The color gradient indicates the adjusted p-value (two-sided Wald test, multiple testing correction with Benjamini & Hochberg method in DESeq2). Individual panels show individual FunCs (1 to 4). Highlighted DEGs were selected for KOs with (**a**) functions involved in lipid accumulation and (**b**) functions involved in beta-oxidation.

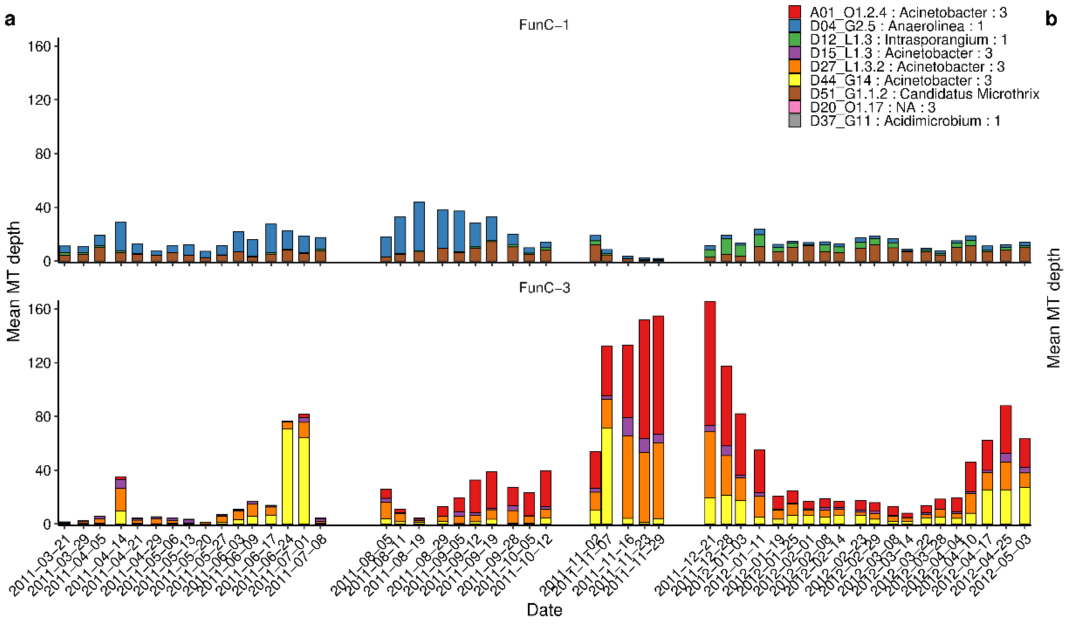

Supplementary Figure 11: Wax-ester synthase/diacylglycerol acyltransferase (WS/DGAT) gene expression levels over time. Only rMAGs with a mean MT-depth of at least 10 in any time-point for genes annotated with KO: K00635 are shown (n=9 rMAGs). In situ (a) and ex situ (b) experiments. Colors indicate rMAG assignment in the format: “rMAG ID : Genus level assignment: FunC-ID”.

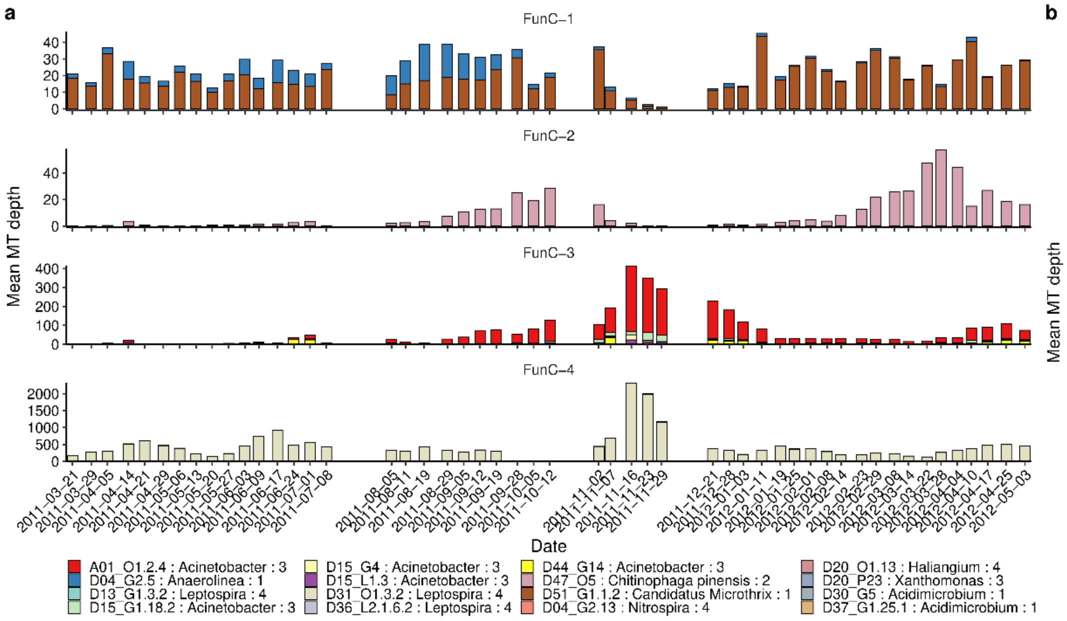

Supplementary Figure 12: TAG lipase gene expression levels over time. Only rMAGs with a mean MT-depth of at least 10 in any time-point for genes annotated with KO:K01046 are shown (n=16 rMAGs). In situ (a) and ex situ (b) experiments. Colors indicate rMAG assignment in the format: “rMAG ID : Genus level assignment: FunC-ID”.

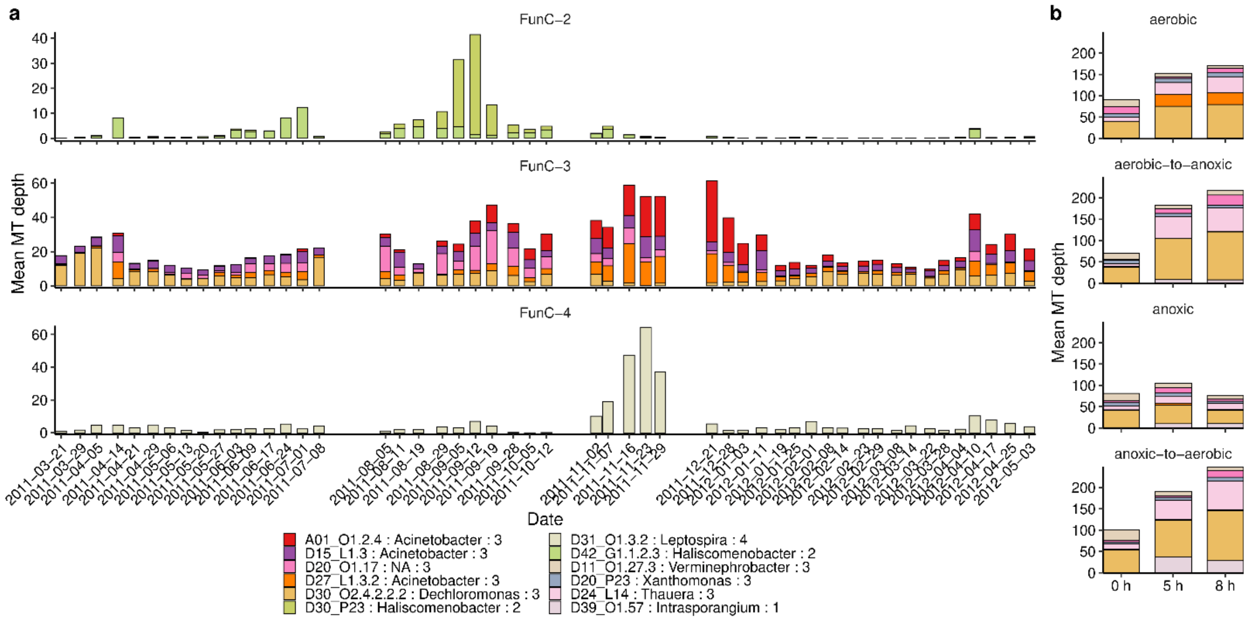

Supplementary Figure 13: PHA synthase gene expression levels over time. Only rMAGs with a mean MT-depth of at least 10 in any time-point for genes annotated with KO:K03821 are shown (n=12 rMAGs). In situ (a) and ex situ (b) experiments. Colors indicate rMAG assignment in the format: "rMAG ID : Genus level assignment: FunC-ID".

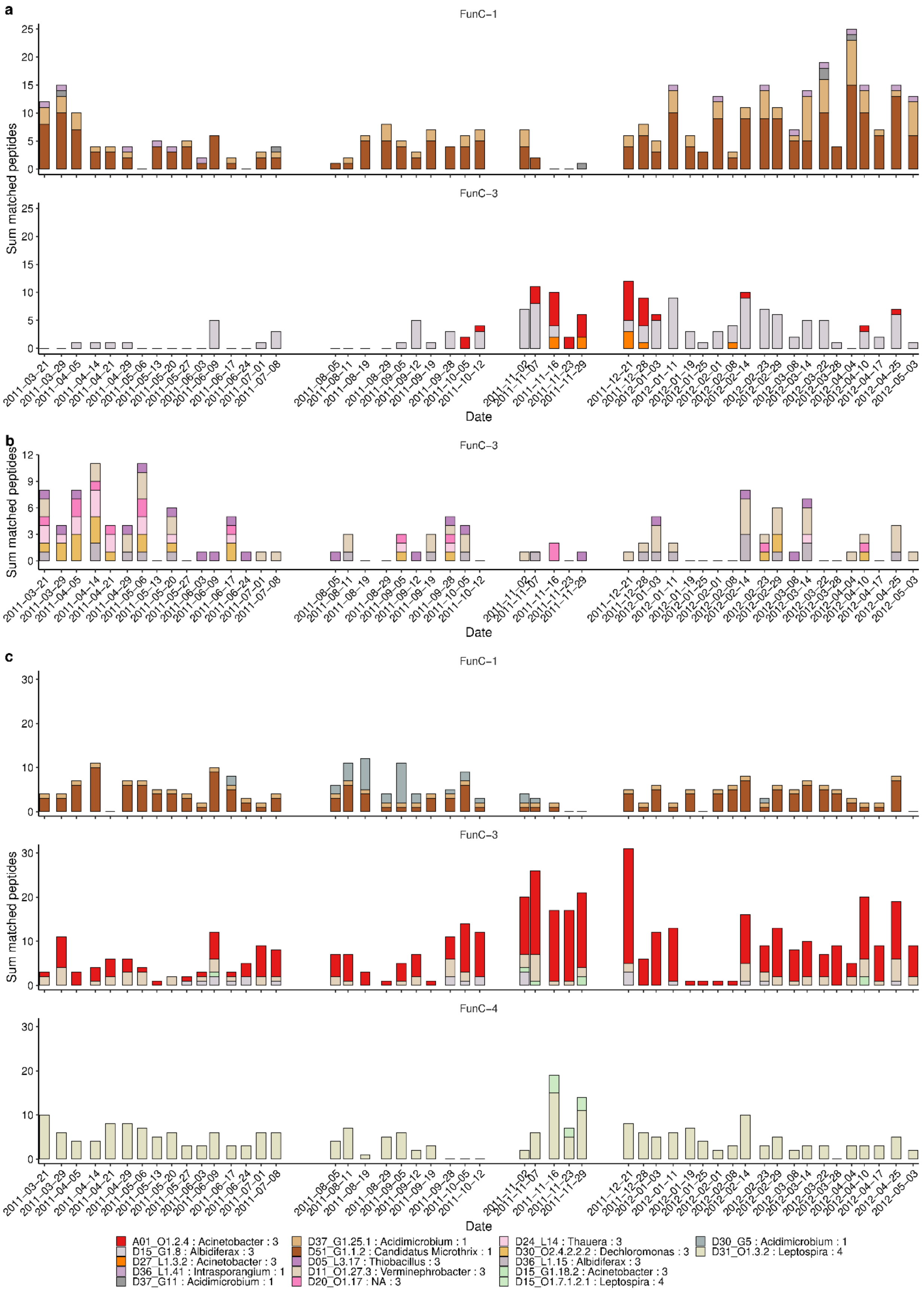
a
FunC−1
FunC−3
Sum matched peptides
Date
b
FunC−3
Sum matched peptides
Date
c
FunC−1
FunC−3
FunC−4
Sum matched peptides
Date
2011−03−21
2011−03−29
2011−04−05
2011−04−14
2011−04−21
2011−04−29
2011−05−06
2011−05−13
2011−05−20
2011−05−27
2011−06−03
2011−06−09
2011−06−17
2011−06−24
2011−07−01
2011−07−08
2011−08−05
2011−08−11
2011−08−19
2011−08−29
2011−09−05
2011−09−12
2011−09−19
2011−09−28
2011−10−05
2011−10−12
2011−11−02
2011−11−07
2011−11−16
2011−11−23
2011−11−29
2011−12−21
2011−12−28
2012−01−03
2012−01−11
2012−01−19
2012−01−25
2012−02−01
2012−02−08
2012−02−14
2012−02−23
2012−02−29
2012−03−08
2012−03−14
2012−03−22
2012−03−28
2012−04−04
2012−04−10
2012−04−17
2012−04−25
2012−05−03
A01_O1.2.4 : Acinetobacter : 3
D15_G1.8 : Albidiferax : 3
D27_L1.3.2 : Acinetobacter : 3
D36_L1.41 : Intrasporangium : 1
D37_G11 : Acidimicrobium : 1
D37_G1.25.1 : Acidimicrobium : 1
D51_G1.1.2 : Candidatus Microthrix : 1
D05_L3.17 : Thiobacillus : 3
D11_O1.27.3 : Verminephrobacter : 3
D20_O1.17 : NA : 3
D24_L14 : Thauera : 3
D30_O2.4.2.2.2 : Dechloromonas : 3
D36_L1.15 : Albidiferax : 3
D15_G1.18.2 : Acinetobacter : 3
D15_O1.7.1.2.1 : Leptospira : 4
D30_G5 : Acidimicrobium : 1
D31_O1.3.2 : Leptospira : 4

Supplementary Figure 14: Proteomics of WS/DGAT, PHA synthase, and TAG lipase measured in the *in situ* time-series. Spectral counts of assigned peptides to genes annotated as WS/DGAT (K00635; **a**), PHA synthase (K03821; **b**), and TAG lipase (K01046; **c**) in the respective rMAG are shown for selected rMAGs with at least 5 assigned spectral counts over all time-points (n=17 rMAGs). Colors indicate rMAG assignment and panels are separated according to FunC assignment.

Supplementary Note 1: Expression of key functions involved in lipid metabolism over time

Using the integrated meta-omics analysis of in situ and ex situ samples, we revealed distinct, condition-specific responses of the constituent populations, notably in relation to lipid accumulation. To better understand how the individual populations' contributions to lipid metabolism change over time and to resolve key functionalities, we tracked the expression levels of individual genes central to lipid metabolism in the short-term and long-term time-series. Specifically, we focused on TAG lipases (K01046), DGAT/WS (K00635), and PHB synthase (K03821).

The expression of TAG lipases was apparent in all FunCs, but especially pronounced in FunC-3 and FunC-4 (Supplementary Fig. 12a). rMAG D31_O1.3.2 (*Leptospira*, FunC4) showed particularly high expression of TAG lipases in November 2011. This, in combination with the encoding of functions for beta-oxidation, indicates a capability to catabolize LCFAs by this population rather than lipid accumulation. High gene expression levels were also observed at 0h for all the ex situ experiments (Supplementary Fig. 12b), but these levels decreased in the following 5h and 8h time-points after the addition of oleic acid. This was also the case for rMAG D04_G2.5 (*Anaerolinea*, FunC1) except for the anoxic condition where lipase expression remained constant. In the in situ experiments, increased expression of lipases was apparent for D04_G2.5 in late summer and the early autumn time-points. We observed contiguous time intervals with similarly increased MT depth for different populations, including a *Chitinophaga* rMAG with peaks in lipase expression in autumn 2011 and spring 2012 (Supplementary Fig. 12). For *Microthrix* D51_G1.1.2 expression levels remained more stable, except for the decline in November 2011. Overall, levels were slightly elevated in winter compared to spring and summer time-points. *Microthrix* also showed a constant high expression of TAG lipases in the short-term experiments. We observed similar trends at the MP level, albeit with a higher variance (Supplementary Fig. 14c). Although, the different populations expressed TAG lipase at distinct magnitudes, intervals of peak expression seemed to be driven by niche complementarity in overall lipase activity. At a functional level, there exists competition in relation to distinct metabolic strategies for lipids, namely catabolism (performed by *Leptospira* spp., *Anaerolinea* spp., and

Chitinophaga spp.) and storage (performed by *Microthrix* spp.). To favour lipid storage, niches should therefore be established which favour lipid accumulation.

Characteristically, *Microthrix* D51_G1.1.2 encoded several DGAT copies highlighting its importance for TAG accumulation[1]. DGAT was expressed throughout most of the time-series (Supplementary Fig. 11) but was elevated in winter, particularly in the MP data (Supplementary Fig. 14a). In the short-term experiments, *Microthrix* showed the highest expression levels for DGAT under aerobic conditions, which was not expected based on previous findings[2]. We observed the opposite trend, i.e., high DGAT expression under anoxic conditions, for *Intrasporangium* (D12_L1.3) and *Acidimicrobium* (D37_G11) of the same FunC (Supplementary Fig. 11b). *Anaerolinea* D04_G2.5 exhibited reduced expression levels for DGAT in winter (Supplementary Fig. 11a) as well as in the short-term incubations in the presence of oxygen (Supplementary Fig. 11b). While few *Acinetobacter* associated rMAGs were covered at relevant MT or MG depth in the short-term experiments, rMAG D44_G14 appeared to upregulate DGAT/WS at 8h in all but the anoxic condition (Supplementary Fig. 11b). This points towards a swift response to elevated LCFA levels for this population, evidenced by individual spikes in gene expression observed in the long-term time-series for this rMAG. DGAT/WS expression was also pronounced in other *Acinetobacter* rMAGs with a gradual increase in MT depth towards late autumn 2011 in A01_O1.2.4 (Supplementary Fig. 11a). The resolved expression levels point towards an increased role for lipid accumulation in these populations in late autumn. However, an increased accumulation of wax-esters also appears plausible based on earlier characterisations of *Acinetobacter*[3].

Acinetobacter-associated rMAGs accumulated PHA, as evidenced by the expression levels of PHB synthase (Supplementary Fig. 13). While the enzyme was not detected at the MP level (Supplementary Fig. 14), a signal was detected at the MT level for distinct rMAGs, mainly in late November. Other rMAGs of FunC3 such as *Dechloromonas* D30_O2.4.2.2.2 exhibited PHB synthase expression consistently throughout the time-series except for late November (Supplementary Fig. 13). Interestingly, this population as well as *Thaurea* showed increasing PHB synthase MT depth for the aerobic conditions in the short-term experiments, while an *Acinetobacter* population could also be detected at higher MT depth only in the aerobic experiment.

Supplementary References

1. Sheik, A. R., Muller, E. E. L. & Wilmes, P. A hundred years of activated sludge: time for a rethink. *Front. Microbiol.* **5**, 1–7 (2014).
2. May, R. M. & Arthur, R. H. M. Niche overlap as a function of environmental variability. *Proc. Natl. Acad. Sci.* **69**, 1109–1113 (1972).
3. Alvarez, H. M. Triacylglycerol and wax ester-accumulating machinery in prokaryotes. *Biochimie* **120**, 28–39 (2016).
