# Supplementary material for: Integration of time-series meta-omics data reveals how microbial ecosystems respond to disturbance
Source: Nat Commun. 2020 Oct 19;11:5281. doi: 10.1038/s41467-020-19006-2 (PMC7572474; doi:10.1038/s41467-020-19006-2)
Supplement: Supplementary file 3 — Description of Additional Supplementary Files [file 41467_2020_19006_MOESM3_ESM.pdf]

Description of Additional Supplementary Files

File Name: Supplementary Data 1
Description:

- Overview of IMP analysis:
  Overview of MT and MG read counts for different steps of the analysis of the 53 in situ samples.

File Name: Supplementary Data 2
Description:

- Summary rMAGs:
  Characterisation of the set of 78 filtered rMAGs mainly utilized in the analysis. Reporting of checkM results, FunC assignment, and taxonomy assignments.
- MIMAG reporting:
  MIMAG reporting for all MAGs generated in the analysis including assembly statistics, MAG quality estimates, predicted tRNAs and rRNAs
- rMAG stability MG:
  Inferred population stability based on the relative MG abundance of 78 rMAGs.
- rMAG stability MT:
  Inferred population stability based on the relative MT abundance of 78 rMAGs.

File Name: Supplementary Data 3
Description:

- Abiotic factors - final values:
  Values for all time-points derived from metabolomics measurements, metabolite ratios, or physico-chemical parameter measurements in long format. Including normalized values and categorization of the parameters.
- BNP_raw: Initial intensities for derivates from the meta-metabolomic measurements of intracellular (biomass) non-polar metabolites.
- BP_raw:
  Initial intensities for derivates from the meta-metabolomic measurements of intracellular (biomass) polar metabolites.
- SNP_raw:
  Initial intensities for derivates from the meta-metabolomic measurements of extracellular (supernatant) non-polar metabolites.
- SP_raw:
  Initial intensities for derivates from the meta-metabolomic measurements of extracellular (supernatant) polar metabolites.

- BNP_poolnorm:
  Pool-normalised (see methods) intensities of derivates from the meta-metabolomic measurements of intracellular (biomass) non-polar metabolites.
- BP_ poolnorm:
  Pool-normalised (see methods) intensities of derivates from the meta-metabolomic measurements of intracellular (biomass) polar metabolites.
- SNP_ poolnorm:
  Pool-normalised (see methods) intensities of derivates from the meta-metabolomic measurements of extracellular (supernatant) non-polar metabolites
- SP_ poolnorm:
  Pool-normalised (see methods) intensities of derivates from the meta-metabolomic measurements of extracellular (supernatant) polar metabolites
- BNP_seq:
  Measurement sequence of samples with technical replicates, pools, and blanks for the meta-metabolomic measurements of intracellular (biomass) non-polar metabolites.
- BP_seq:
  Measurement sequence of samples with technical replicates, pools, and blanks for the meta-metabolomic measurements of intracellular (biomass) polar metabolites.
- SNP_seq:
  Measurement sequence of samples with technical replicates, pools, and blanks for the meta-metabolomic measurements of extracellular (supernatant) non-polar metabolites.
- SP_seq:
  Measurement sequence of samples with technical replicates, pools, and blanks for the meta-metabolomic measurements of extracellular (supernatant) polar metabolites.
- Derivate_to_Database_Links:
  Manual assignment of derivate IDs to metabolites IDs from the KEGG compound database and CHEBI. These levels were used to select intensity values from specific derivates or group metabolites according to a metabolite class.

File Name: Supplementary Data 4
Description:

- Manual_PC_collection:
  Measurements of physico-chemical parameters during sampling.
- Manual_processed:
  Corrected measurements of the collection of physico-chemical parameters during sampling, replacement of missing values with NA.
- Online_collection:
  Measurements as received from the WWTP operators for values for the

anoxic tank from which samples were taken. Values correspond to averages of 2h intervals

- Online_processed_daily_avgs:
  Daily averages for the measurements in “Online_Collection”.
- Online_all_processed_2h:
  Measurements as received from the WWTP operators for additional parameters at different locations in the WWTP. Values correspond to 2h intervals.

File Name: Supplementary Data 5
Description:

- S5-KO_enrichment_funcs:
  Enrichment statistics for KO categories within the FunCs.
  Column “rMAGs within FunC with KO” corresponds to the number of rMAGs in a FunC in which the KO can be detected.
  Column “Total number of rMAGs with KO” refers to the number of occurences in rMAGs in all FunCs.
  Rows can be duplicated if a KO can be assigned to multiple pathways (“Pwy_name”)
- S5-KO_ratios_funcs:
  Ratios of KO occurrences within the FunCs as additional information to interpret the enrichment data.
  Column “count_perfunc” gives the number of times the KO occurred within rMAGs of the respective FunC.
  Column “presence_rMAGs” gives the number of rMAGs associated to the respective FunC in which the KO occurred.
  Column “Freq_KOs” denotes in how many rMAGs the respective KO has been detected.
  Column Freq_rMAGs” lists the number of rMAGs per FunC.

File Name: Supplementary Data 6
Description:

- S6a-clusters_with_microthrix_D5:
  Overview of the number time-points in which a KO is considered active (MT/MG ratios > 1 or evidence on the MP level, see methods) within at least 70% of rMAGs that fall in the same cluster of expression profiles as the rMAG D51_G1.1.2 that reflects the most abundant *Microthrix* rMAG.
- S6b-clusters_with_acinetob._A01:
  Overview of the number time-points in which a KO is considered active (MT/MG ratios > 1 or evidence on the MP level, see methods) within at least 70% of rMAGs that fall in the same cluster of expression profiles as the rMAG A01_G1.2.4 that reflects the most abundant *Acinetobacter* rMAG.
