# Supplementary material for: Integration of time-series meta-omics data reveals how microbial ecosystems respond to disturbance
Source: Nat Commun. 2020 Oct 19;11:5281. doi: 10.1038/s41467-020-19006-2 (PMC7572474; doi:10.1038/s41467-020-19006-2)
Supplement: Supplementary file 10 — Reporting Summary [file 41467_2020_19006_MOESM10_ESM.pdf]

nature research

Corresponding author(s): Paul Wilmes

Last updated by author(s): Sep 7, 2020

Reporting Summary

Nature Research wishes to improve the reproducibility of the work that we publish. This form provides structure for consistency and transparency in reporting. For further information on Nature Research policies, see our Editorial Policies and the Editorial Policy Checklist.

Statistics

For all statistical analyses, confirm that the following items are present in the figure legend, table legend, main text, or Methods section.

n/a	Confirmed	
☐	☒	The exact sample size (*n*) for each experimental group/condition, given as a discrete number and unit of measurement
☐	☒	A statement on whether measurements were taken from distinct samples or whether the same sample was measured repeatedly
☐	☒	The statistical test(s) used AND whether they are one- or two-sided<br>*Only common tests should be described solely by name; describe more complex techniques in the Methods section.*
☐	☒	A description of all covariates tested
☐	☒	A description of any assumptions or corrections, such as tests of normality and adjustment for multiple comparisons
☐	☒	A full description of the statistical parameters including central tendency (e.g. means) or other basic estimates (e.g. regression coefficient) AND variation (e.g. standard deviation) or associated estimates of uncertainty (e.g. confidence intervals)
☐	☒	For null hypothesis testing, the test statistic (e.g. *F*, *t*, *r*) with confidence intervals, effect sizes, degrees of freedom and *P* value noted<br>*Give P values as exact values whenever suitable.*
☒	☐	For Bayesian analysis, information on the choice of priors and Markov chain Monte Carlo settings
☒	☐	For hierarchical and complex designs, identification of the appropriate level for tests and full reporting of outcomes
☐	☒	Estimates of effect sizes (e.g. Cohen's *d*, Pearson's *r*), indicating how they were calculated

Our web collection on statistics for biologists contains articles on many of the points above.

Software and code

Policy information about availability of computer code

Data collection	Basecalling of metagenomic (MG) and metatranscriptomic (MT) data was processed using commercial software bundled within Illumina sequencing platforms to generate raw FASTQ data. Raw metaproteomic (MP) mass spectra were acquired using commercial software from ThermoFischer Scientific.<br><br>This work represents part of a larger ongoing multi-annual project. Please refer to previous publications for detailed information on NGS and mass spectrometry platforms and the associated software for those platforms:<br>https://doi.org/10.1038/ismej.2012.72<br>https://doi.org/10.1038/ncomms6603<br>https://doi.org/10.1038/npjbiofilms.2015.7<br>https://doi.org/10.1186/s40793-017-0274-y
Data analysis	Software used:<br>R3.4.4 and R3.6.1.<br>IMP 1.3<br>MEGAHIT 1.0.6<br>dRep 0.5.4_SHAMANFIX<br>checkM 1.0.7<br>bwa 0.7.9a<br>MetaboliteDetector Ver. 2.5<br>Prokka 1.11<br>prodigal 2.60<br>Graph2Pro

FragGeneScan
MS/GF+
HMMer 1.12b
Aragorn 1.2.38
MSconvert 3.0
barrnap 0.9
AMPHORA2 2.0
sourmash-lca 2.0.01a1
featureCounts (version number not recorded)
DESeq2 1.18.1
mash 2.2.2
vegan 2.5-5
tidyverse 1.2.1
dbscan 1.1-3
imputeTS 3.1
seqinr v.3.6.169

Data analysis code has been deposited in public repositories.
The code for genome reconstruction and dereplication is available at: https://git-r3lab.uni.lu/shaman.narayanasamy/LAO-time-series .
The code for processing and analysis of the meta-omics data, as well as for additional analyses and generation of plots for main and supplemental figures is available at: https://git-r3lab.uni.lu/malte.herold/laots_niche_ecology_analysis .

For manuscripts utilizing custom algorithms or software that are central to the research but not yet described in published literature, software must be made available to editors and reviewers. We strongly encourage code deposition in a community repository (e.g. GitHub). See the Nature Research guidelines for submitting code & software for further information.

Data

Policy information about availability of data

All manuscripts must include a data availability statement. This statement should provide the following information, where applicable:
- Accession codes, unique identifiers, or web links for publicly available datasets
- A list of figures that have associated raw data
- A description of any restrictions on data availability

Meta-omics data from five individual time-points have been previously published and respective references are provided in the manuscript.
The MG and MT FASTQ files and the sample-wise MT- and co-assembly contigs are available on NCBI BioProject PRJNA230567. MP data has been deposited in the PRIDE database under the accession number PXD013655.
Raw metabolomics data is available at metabolights under the accession MTBLS2021, while processed intensities after identification are provided with this manuscript (Supplementary Data 3). Similarly, physico-chemical parameters are provided with this manuscript (Supplementary Data 4).
Processed and intermediary data files from the combined multi-omic analyses are available at the following archive:
doi:10.5281/zenodo.3961685
Source data for figures are provided with this paper.
External databases were used in this study: KEGG (https://www.genome.jp/kegg/), CHEBI (https://www.ebi.ac.uk/chebi/).

Field-specific reporting

Please select the one below that is the best fit for your research. If you are not sure, read the appropriate sections before making your selection.

☐ Life sciences ☐ Behavioural & social sciences ☒ Ecological, evolutionary & environmental sciences

For a reference copy of the document with all sections, see nature.com/documents/nr-reporting-summary-flat.pdf

Ecological, evolutionary & environmental sciences study design

All studies must disclose on these points even when the disclosure is negative.

Study description	We studied the composition and function of an oleaginous microbial community at the air-water interface of an anoxic tank in a wastewater treatment plant. Samples were taken in weekly intervals over a 1.5 year time period. 51 in situ time-series samples were analyzed (metagenomic, metatranscriptomic, metaproteomic, meta-metabolomic data), as well as 2 previously published in situ samples (metagenomic, metatranscriptomic data). 12 ex situ samples were analysed (metagenomic, metatranscriptomic data).
Research sample	Individual floating sludge islets from the surface of the anoxic tank of the Schifflange biological wastewater treatment plant were sampled due to their richness in lipid accumulating organisms. They were then subjected to a concomitant biomolecular extraction of DNA, RNA and proteins, and high-throughput measurements to obtain metagenomic, metatranscriptomic and metaproteomic datasets for downstream analyses.<br>Additionally, biomass from bioreactor experiments seeded with sludge samples from the same anoxic tank were sampled as a closest representative of the in situ community allowing for targeted perturbation. These samples were subjected to a concomitant biomolecular extraction of DNA and RNA to obtain metagenomic and metatranscriptomic datasets.<br>All samples represent individual time-points and conditions.

Sampling strategy	In situ samples:<br>Samples were collected from Schifflange biological wastewater treatment plant (Esch-sur-Alzette, Luxembourg; 49°30′48.29″N; 6°1′ 4.53″E). Individual floating sludge islets were collected from the same spot of the anoxic tank, along with physico-chemical parameters of the water, i.e. pH, temperature, conductivity, oxygen.<br>Two initial samples were collected on 2010-10-04 and 2011-01-25 in the context of previously published work (https://doi.org/10.1038/ncomms6603 and https://doi.org/10.1038/npjbiofilms.2015.7). More frequent sampling was performed from 2011-03-21 to 2012-05-03, of which data from three samples (2011-10-05, 2011-10-05 and 2012-01-11) have been previously published (https://doi.org/10.1038/ncomms6603). A total of 53 samples were collected over a period of 578 days. The mean sample frequency was 8 days (SD=16 days). The sampling procedure was designed to span at least one entire annual seasonal cycle (i.e. winter, spring, summer, autumn) while the sampling frequency corresponded to the doubling time of the dominant bacterial population of approximately 8 days, thus representing an approximate generational time scale.<br>Sampling was performed by Laura A. Lebrun and Emilie E.L. Muller.<br>This work represents part of a larger ongoing multi-annual project. Thus, all the samples were subjected to the same experimental protocols. Please refer to detailed methods on sampling procedures in previous publications:<br>https://doi.org/10.1038/ncomms6603<br>https://doi.org/10.1038/npjbiofilms.2015.7<br><br>Ex situ samples:<br>Samples from sludge mixed with artificial wastewater were subjected to targeted aerobic, anerobic, or alternating conditions after addition of oleic acid and nutrients. Samples for biomolecular extraction of DNA and RNA were taken at 0h, 5h, 8h after a substrate pulse to capture a transcriptional response, while also confirming that the community composition remained unaltered during this time-frame.<br>Sampling and biomolecular extractions on these samples were performed by Abdul R. Sheik.<br>Experimental setup and treatments subjected to the different samples are detailed in a prior study:<br>https://doi.org/10.1038/ismej.2015.181
Data collection	Laura A. Lebrun and Emilie E.L. Muller performed the concomitant biomolecular extractions resulting in fractions of DNA, RNA, proteins and metabolites for each in situ sample.<br>Nathan D. Hicks, Lance B. Price, John D. Gillece, James M. Schupp and Paul S. Keim performed the DNA and RNA library preparation and next-generation sequencing (NGS) to obtain metagenomic and metatranscriptomic data of the in situ samples on an Illumina Genome Analyser (GA) IIx instrument.<br>Irina Bessarab and Rohan B.H. Williams performed the DNA and RNA library preparation and NGS to obtain metagenomic and metatranscriptomic data of the ex situ samples on an Illumina HiSeq2500 instrument.<br><br>Michael R. Hoopmann and Robert L. Moritz performed the mass-spectrometry measurements of the protein fractions on an LTQ-Orbitrap Elite (ThermoFisher Scientific) instrument.<br>Christian Jäger prepared and performed the metabolomic measurements on an Agilent 7890A GC coupled to an Agilent 5975C inert XL Mass Selective Detector (Agilent Technologies).<br>This work represents part of a larger ongoing multi-annual project. For additional detailed information and descriptions about data collection, experimental protocols, experimental kit versions, DNA and RNA library preparation, proteomic sample preparation, high-throughput platforms, please refer to the following articles:<br>https://doi.org/10.1038/ismej.2012.72<br>https://doi.org/10.1038/ncomms6603<br>https://doi.org/10.1038/npjbiofilms.2015.7<br>https://doi.org/10.1186/s40793-017-0274-y
Timing and spatial scale	Individual floating sludge islets within anoxic tank number one of the Schifflange BWWT plant (Esch-sur-Alzette, Luxembourg; 49° 30'48.29"N; 6°1'4.53"E) were sampled always on the same spot. Sampling was carried out from 2010-10-04 to 2012-05-03. Two samples were collected on 2010-10-04 and 2011-01-25, to determine the sequencing conditions and the microbial diversity and was published in previous work. Subsequently, samples were collected on a weekly basis from 2011-03-21 to 2012-05-03, which approximately corresponds to the generational time scale of the sludge of eight days. The lack of samples in periods; from 2011-07-08 to 2011-08-05, from 2011-10-12 to 2011-11-02, and from 2011-11-20 to 2012-12-21 are due to absence of foaming islets as consequence of (i) heavy or continued rain and/or (ii) natural decrease of foam during summer and autumn seasons.<br>The sampling procedure was designed to span at least one entire annual seasonal cycle (i.e. winter, spring, summer, autumn).
Data exclusions	No data were excluded from the analysis.
Reproducibility	Experimental procedures adhered to previously published protocols. Open source software was used in all the computational analyses. All custom scripts and commands are available within GitLab repositories. Wherever applicable, the software versions are reported in "Methods and Material" within the manuscript.
Randomization	Samples collected from 2011-03-21 to 2012-05-03 were randomized before biomolecular extractions. The biomolecular fractions were further randomized prior to the high-throughput measurements.<br>The two initial samples, collected on 2010-10-04 and 2011-01-25, were not included within the aforementioned randomization procedure(s) as they were collected in the context of previous work (https://doi.org/10.1038/ncomms6603 and https://doi.org/10.1038/npjbiofilms.2015.7) and were used to pilot the experimental protocols which was conducted prior to the higher frequency sampling (i.e. from 2011-03-21 to 2012-05-03).
Blinding	Blinding is not applicable in this study as it did not involve human subjects, but samples from a biotechnological system (municipal wastewater treatment plant).

Did the study involve field work? ☒ Yes ☐ No

Field work, collection and transport

Field conditions	Samples were taken from an open anoxic activated sludge tank of a wastewater treatment plant.<br>Field work was subjected to climatic conditions including seasonal variation of temperature and rainfall.<br>To account for varying conditions during field work, metadata, e.g., air temperature, was recorded and is available as Supplementary Data.<br>No sampling occurred in case of absence of foaming islets, as consequence of (i) heavy or continued rain and/or (ii) natural decrease of foam during summer and autumn seasons.
Location	SIVEC, rue de Bergem, Schifflange, Luxembourg; 49°30'48.29"N; 6°1'4.53"E
Access & import/export	Access was granted to the research personnel based on agreement between the principal investigator, Prof. Paul Wilmes (on behalf of the research institution), and the wastewater treatment facility management (Mr. Bissen and Mr. Di Pentima) from the Syndicat Intercommunal a Vocation Ecologique (SIVEC), Schifflange, Luxembourg. All research personnel are informally introduced to the management and personnel of the facility prior to conducting any work. Research personnel were not provided with keys or electronic access cards, and thus could only enter the premises upon the permission of personnel at the entrance of the facility.
Disturbance	Sampling had a minimum-to-no impact on the operations of the wastewater treatment facility. The work of the researchers did not require (complete or partial) shutdown or any operational disruption of the facility. Sampling was performed by the research personnel (Hugo Roume, Abdul R. Sheik, Paul Wilmes, Emilie E.L. Muller and Laura A. Lebrun) without any involvement of the staff of the facility. Research personnel either brought their own equipment or used equipment from the site, which was dedicated to them, thus not hindering any operations or personnel within the facility. Researchers could access operational readings (e.g. temperature, inflow, outflow, etc.) of the facility directly via a dedicated web portal of the facility using login credentials provided by the facility management.

Reporting for specific materials, systems and methods

We require information from authors about some types of materials, experimental systems and methods used in many studies. Here, indicate whether each material, system or method listed is relevant to your study. If you are not sure if a list item applies to your research, read the appropriate section before selecting a response.

Materials & experimental systems

n/a	Involved in the study	
☒	☐	Antibodies
☒	☐	Eukaryotic cell lines
☒	☐	Palaeontology and archaeology
☒	☐	Animals and other organisms
☒	☐	Human research participants
☒	☐	Clinical data
☒	☐	Dual use research of concern

Methods

n/a	Involved in the study	
☒	☐	ChIP-seq
☒	☐	Flow cytometry
☒	☐	MRI-based neuroimaging
